# Supplementary material for: Agreement between physician- and patient-reported Canadian cardiovascular society scores among patients undergoing elective coronary angiography–The CATS study
Source: PLoS One. 2023 Oct 13;18(10):e0292058. doi: 10.1371/journal.pone.0292058 (PMC10575503; doi:10.1371/journal.pone.0292058)
Supplement: S1 File — (PDF) [file pone.0292058.s001.pdf]

S1 File. The CCS questionnaire.

CCS class

During exercise, the blood flow of the heart does not increase sufficiently as a result of coronary artery disease. Due to this, the patient experiences symptoms, which may be chest pain, tightness of chest or shortness of breath (which feels different than the normal loss of breath caused by exercise).

The occurrence of chest pain and its severity are measured by the so-called CCS scale. It describes the situations in which chest pain occurs and how much it restricts the normal life.

In the table below, mark the option that best describes the severity of your symptoms.

☐ Normal physical exertion does not cause me chest pain, tightness of chest or shortness of breath. Chest pain, tightness of chest or shortness of breath arise only in a heavy, rapid, or prolonged exertion.

☐ Chest pain, tightness of chest or shortness of breath cause only a slight restriction to my normal activities. Chest pain, tightness of chest or shortness of breath occur during fast walking or stair climbing, moving after a meal, in a cold or windy weather, under intense emotions or during the first hours after waking up. During normal walking, chest pain arises after walking more than two blocks (more than 200 meters) or after climbing two or more flight of stairs.

☐ Chest pain, tightness of chest or shortness of breath cause me a significant restriction to the ordinary activities. In normal circumstances, chest pain, tightness of chest or shortness of breath occur at normal walking pace at the distance of 1-2 blocks (less than 200 meters) or when climbing one flight of stairs.

☐ Physical exertion without chest pain, tightness of chest or shortness of breath is not possible for me. Chest pain, tightness of chest or shortness of breath may arise even when resting.

Reference: Campeau L. Letter: Grading of angina pectoris. Circulation 1976;54:522:523
